# Bariatric Surgery in Obese Women of Reproductive Age Improves Conditions That Underlie Fertility and Pregnancy Outcomes: Retrospective Cohort Study of UK National Bariatric Surgery Registry (NBSR)

**DOI:** 10.1007/s11695-016-2202-4

**Published:** 2016-06-18

**Authors:** Eric Edison, Martin Whyte, Jeremy van Vlymen, Simon Jones, Piers Gatenby, Simon de Lusignan, Jill Shawe

**Affiliations:** 1 Faculty of Health and Medical Sciences, University of Surrey, Guildford, Surrey GU2 7XH UK; 2 Regional Oesophago-Gastric Unit, Royal Surrey County Hospital, Guildford, Surrey GU2 7XX UK

**Keywords:** Obesity, Bariatric surgery, Pregnancy, Fertility, Parturition, Neonatal outcomes, Preconception care

## Abstract

**Background:**

The aims of this study are the following: to describe the female population of reproductive age having bariatric surgery in the UK, to assess the age and ethnicity of women accessing surgery, and to assess the effect of bariatric surgery on factors that underlie fertility and pregnancy outcomes.

**Methods:**

Demographic details, comorbidities, and operative type of women aged 18–45 years were extracted from the National Bariatric Surgery Registry (NBSR). A comparison was made with non-operative cases (aged 18–45 and BMI ≥40 kg/m^2^) from the Health Survey for England (HSE, 2007–2013). Analyses were performed using “R” software.

**Results:**

Data were extracted on 15,222 women from NBSR and 1073 from HSE. Women aged 18–45 comprised 53 % of operations. Non-Caucasians were under-represented in NBSR compared to HSE (10 vs 16 % respectively, *p* < 0.0001). The NBSR group was older than the HSE group—median 38 (IQR 32–42) vs 36 (IQR 30–41) years (Wilcoxon test *p* < 0.0001). Almost one third of women in NBSR had menstrual dysfunction at baseline (33.0 %). BMI fell in the first year postoperatively from 48.2 ± 8.3 to 37.4 ± 7.5 kg/m^2^ (*t* test, *p* < 0.001). From NBSR, in the postoperative period, the prevalence of type 2 diabetes fell by 54 %, polycystic ovarian syndrome by 15 %, and any menstrual dysfunction by 12 %.

**Conclusions:**

Over half of all bariatric procedures are carried out on women of reproductive age. More work is required to provide prompt and equal access across ethnic groups. At least one in three women suffers from menstrual dysfunction at baseline. Bariatric surgery improves factors that underlie fertility and pregnancy outcomes. A prospective study is required to verify these effects.

## Introduction

Obesity and its related comorbidities impair fertility, maternal health during pregnancy, maternal obstetric outcomes, fetal outcomes, and long-term health of the offspring [1]. Obesity-related comorbidities are routinely recorded before and after bariatric surgery in the UK National Bariatric Surgery Registry (NBSR) [2, 3]. The NBSR is a comprehensive, prospective, nationwide analysis of outcomes from bariatric surgery in the UK and Ireland. The comorbidities measured include the following: menstrual dysfunction, polycystic ovarian syndrome (PCOS), type 2 diabetes, dyslipidemia, hypertension, asthma, joint pain, functional status, gastro-esophageal reflux, and sleep disorder. Menstrual dysfunction, as defined in the registry, refers to the presence of amenorrhea, irregular periods, or menorrhagia. Functional status in this context refers to exercise tolerance, deemed impaired if the patient is unable to climb a flight of stairs without stopping.

Irregular ovulation associated with menstrual disorders, including PCOS, directly affects fertility. Insulin resistance, as a component of type 2 diabetes, may also affect fertility [4], and, in addition, the maternal health, fetal outcomes, and long-term health of the offspring are impaired by dysglycemia in pregnancy [5, 6]. Dyslipidemia is associated with obstetric vasculopathies [7]. Hypertension increases the rates of pre-eclampsia and small-for-gestational age [8]. Joint pain, functional status, asthma, gastro-esophageal reflux, and sleep disorders are all common in obesity and can all be exacerbated by pregnancy, impairing the well-being of pregnant women.

Bariatric surgery reduces body weight and is an effective intervention in treating type 2 diabetes in obese patients [9], but its effects on fertility and pregnancy outcomes (including maternal health during pregnancy) are not fully elucidated. It is known that weight loss achieved through lifestyle change may help improve fertility [10], but there are limited data demonstrating that this is the case following BS. The official NBSR reports demonstrate an improvement in all (ten) recorded comorbidities after bariatric surgery, but no subanalyses have been made in women of reproductive age.

The aims of this study are to describe the cohort of females of reproductive age having bariatric surgery in the UK, to assess the age and ethnicity of the cohort who do access surgery, and to assess the effect of bariatric surgery on factors which underlie fertility and pregnancy outcomes.

## Methods

Data were extracted from the UK NBSR for women aged 18–45 years. Baseline data included age, weight, height, date of operation, operation type, and presence of comorbidities. Comparative data from the overall cohort were taken from the NBSR reports 1 and 2 [2, 3]. Follow-up data were extracted up to 12 months after the operation and included weight and the presence of comorbidities. The NBSR records ten comorbidities before and after operation: type 2 diabetes, menstrual dysfunction, polycystic ovarian syndrome, dyslipidemia, hypertension, asthma, joint pain, functional status, gastro-esophageal reflux, and sleep disorder.

In order to compare the prevalence of each comorbidity before and after surgery, those patients with missing follow-up data were initially excluded from analysis. This follows the convention of the official NBSR reports. Sensitivity analyses were then applied, assuming that all of the patients with no follow-up had no change to their status. The prevalences were compared using the chi-squared test.

For comparison to a representative national cohort, data were extracted from the Health Survey for England (HSE), which is an annual national census. Data were collated from 2007 to 2013 inclusive. Women aged 18 to 45 with a BMI ≥40 kg/m^2^ (representing those who may be considered for bariatric surgery by NICE criteria) were selected. From this cohort, the median age was compared to the bariatric surgery cohort using Wilcoxon’s test. The prevalence of type 2 diabetes and the ethnic composition of the groups were compared using a chi-squared test. All analyses were performed using the “R” statistical software.

## Results

### NBSR Cohort at the Time of Surgery

In total, there were 29,010 male and female patients who underwent bariatric surgery to year 2013, of whom 15,222 were women aged 18 to 45 years old (53 %). The numbers and percentages of each operation, with comparison to the overall NBSR cohort, are presented in Table 1. The rates of each operation were no different between the groups. The discrepancy in the number of BPD procedures is because the female data dates back to 2003—all 18 of these operations were performed before 2007.

**Table 1:** Comparison of the type of operations performed on females of reproductive age versus the total bariatric population

	Female, aged 18–45, 2003–2013					NBSR report (financial years ending 2009–2013)				
	Total	Total (%)	Lap/endo	Open	Unspec.	Total	Total (%)	Lap/endo	Open	Unspec.
Roux-and-Y gastric bypass	8281	54.40 %	7462	811	8	12,759	54.43 %	11,692	1049	18
Gastric band	3955	25.98 %	3941	4	10	5764	24.59 %	5746	9	9
Sleeve gastrectomy	2457	16.14 %	2446	8	3	4174	17.81 %	4140	24	10
Gastric balloon	246	1.62 %	245	0	1	406	1.73 %	402	0	4
Other	126	0.83 %	123	3	0	206	0.88 %	193	8	5
Unspecified	128	0.84 %	8	0	120	116	0.49 %	5	0	111
Biliopancreatic diversion	18^a^	0.12 %	1	17	0	1^a^	0.00 %	0	0	1
Duodenal switch	11^b^	0.07 %	10	1	0	13^b^	0.06 %	12	1	0
Total	15,222		14,236	844	142	23,439		22,190	1091	158

^a^Note that the discrepancy between biliopancreatic diversion procedures in the overall NBSR cohort (1) and the selected cohort (18) is because our search returned all operations recorded on the register. All biliopancreatic diversions recorded in this cohort were performed from 2003 to 2007, whereas the NBSR formal report is on procedures from 2008

^b^Four of the DS procedures were performed before 2008

### Comparison of NBSR Patients to HSE Subjects

Collating the HSE data from 2007 to 2013 produced a total cohort of 91,649 subjects. Female subjects, aged 18–45 years old, with a recorded BMI of ≥40 kg/m^2^ totaled 384 subjects. The NBSR cohort had a median age of 38 years (IQR 32–42) and was older than the HSE cohort (median 36 years, IQR 30–41; *p* < 0.0001, Wilcoxon test). Caucasian patients were overrepresented in the NBSR cohort (90 % of NBSR vs 84 % HSE, *p* < 0.0001, chi-squared test, Table 2). Bariatric patients were twice as likely to have type 2 diabetes as HSE subjects (14.7 % NBSR vs 7.4 % HSE, *p* < 0.0001, chi-squared test).

**Table 2:** Comparison of the ethnic composition of the national obese population versus the surgical population

Self-reported ethnicity	UK national population females aged 18–45 years old, BMI ≥40 kg/m^2^	UK females aged 18–45 years old, BMI ≥40 kg/m^2^, undergoing bariatric surgery
African/Afro-Caribbean	7.81 %	4.53 %
Asian	5.73 %	3.54 %
Caucasian	84.1 %	89.9 %
Other	2.34 %	1.99 %

### Follow-Up of NBSR Cohort Following Bariatric Surgery

There were 1107 patients with no follow-up data recorded in the registry, leaving 14,115 women with follow-up data of some kind. However, there was some inconsistency in recording of comorbidities. For instance, the number of patients with complete data for hypertension was 8668 (see Table 3). Body weight and the presence or absence of comorbidities were recorded at variable time points over the 12-month postoperative period.

**Table 3:** The rate of obesity-related comorbidities at baseline

Comorbidity	Number of cases	Total number of patients (with complete data)	Prevalence
Menstrual dysfunction	2355	7134	33.0 %
PCOS	1298	8250	15.7 %
Type 2 diabetes	1266	8609	14.7 %
Dyslipidemia	970	8558	11.3 %
Hypertension	1736	8623	20.1 %
Sleep disorder	985	8201	12.0 %
Impaired functional status	5607	8394	66.8 %
Joint pain	3707	8528	43.5 %
Asthma	1742	8623	20.2 %
Gastro-esophageal reflux	1560	7415	21.0 %

**Fig. 1:**
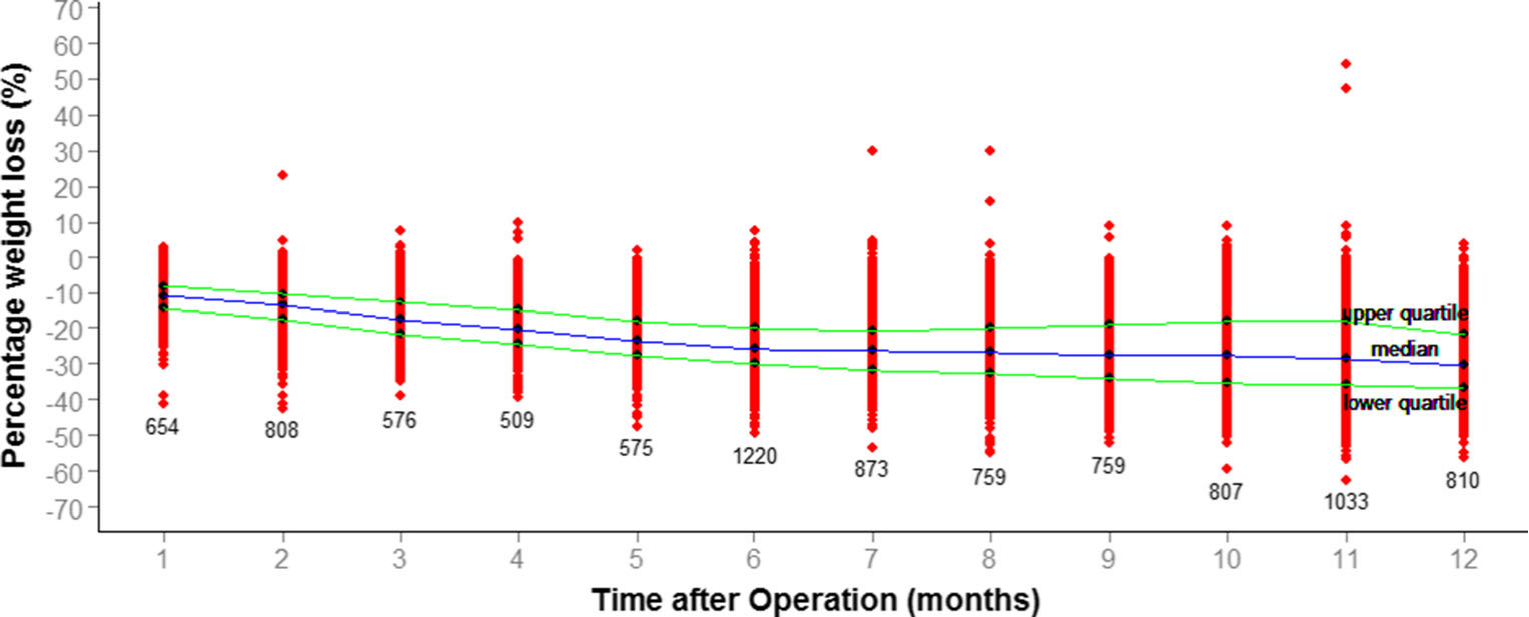
Change in BMI based on the month of follow-up. The mean BMI at baseline is shown by *dot* and *error bars* at 0 months. The mean BMI recorded at each month of follow-up with *error bars*. One point per patient is shown on graph

The mean BMI fell in the first year postoperatively from 48.2 ± 8.3 to 37.4 ± 7.5 kg/m^2^ (*p* < 0.001, *t* test). As this was recorded at variable time points, this is represented graphically (Fig. 1).

**Fig. 2:**
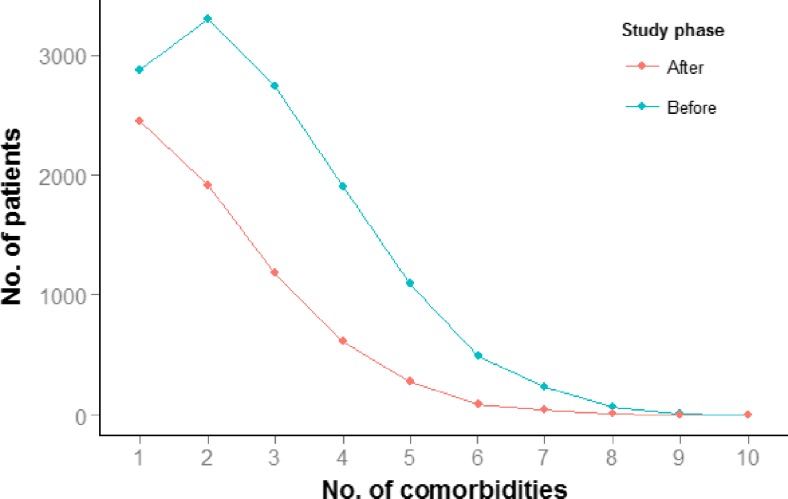
Total number of comorbidities before and after operation

Table 3 demonstrates the numbers of each comorbidity at baseline, following the exclusion of cases with missing data. Surgery led to a reduction in the numbers of each comorbidity (Table 4). These findings remained valid after the sensitivity analysis (i.e., assuming no change in status to patients without follow-up). Of women with menstrual dysfunction presurgery, 12 % of patients normalized menstrual function postoperatively. Similarly, of women with PCOS preoperatively, 15 % were classed as no longer having the syndrome postoperatively. Type 2 diabetes showed the greatest improvement with 54 % of those patients who had diabetes preoperatively achieving a non-diabetic state postoperatively. The mean total number of comorbidities per patient fell from 2.36 to 0.96 (*p* < 0.05, *t* test, Fig. 2).

**Table 4:** Improvement in the rate of obesity-related comorbidities after operation

Comorbidity	Number of patients with comorbidity before surgery	Number of patients with comorbidity after surgery	Proportion improved after surgery	*p* value
Menstrual dysfunction	2355	2063	12.4 %	*p* < 0.0001
PCOS	1298	1106	14.8 %	*p* < 0.0001
Type 2 diabetes	1266	587	53.6 %	*p* < 0.0001
Dyslipidemia	970	497	48.8 %	*p* < 0.0001
Hypertension	1736	1013	41.7 %	*p* < 0.0001
Sleep disorder	985	561	43.1 %	*p* < 0.0001
Impaired functional status	5607	2967	47.1 %	*p* < 0.0001
Joint pain	3707	2605	29.7 %	*p* < 0.0001
Asthma	1742	1222	29.9 %	*p* < 0.0001
Gastro-esophageal reflux	1560	1312	15.9 %	*p* < 0.0001

**Fig. 3:**
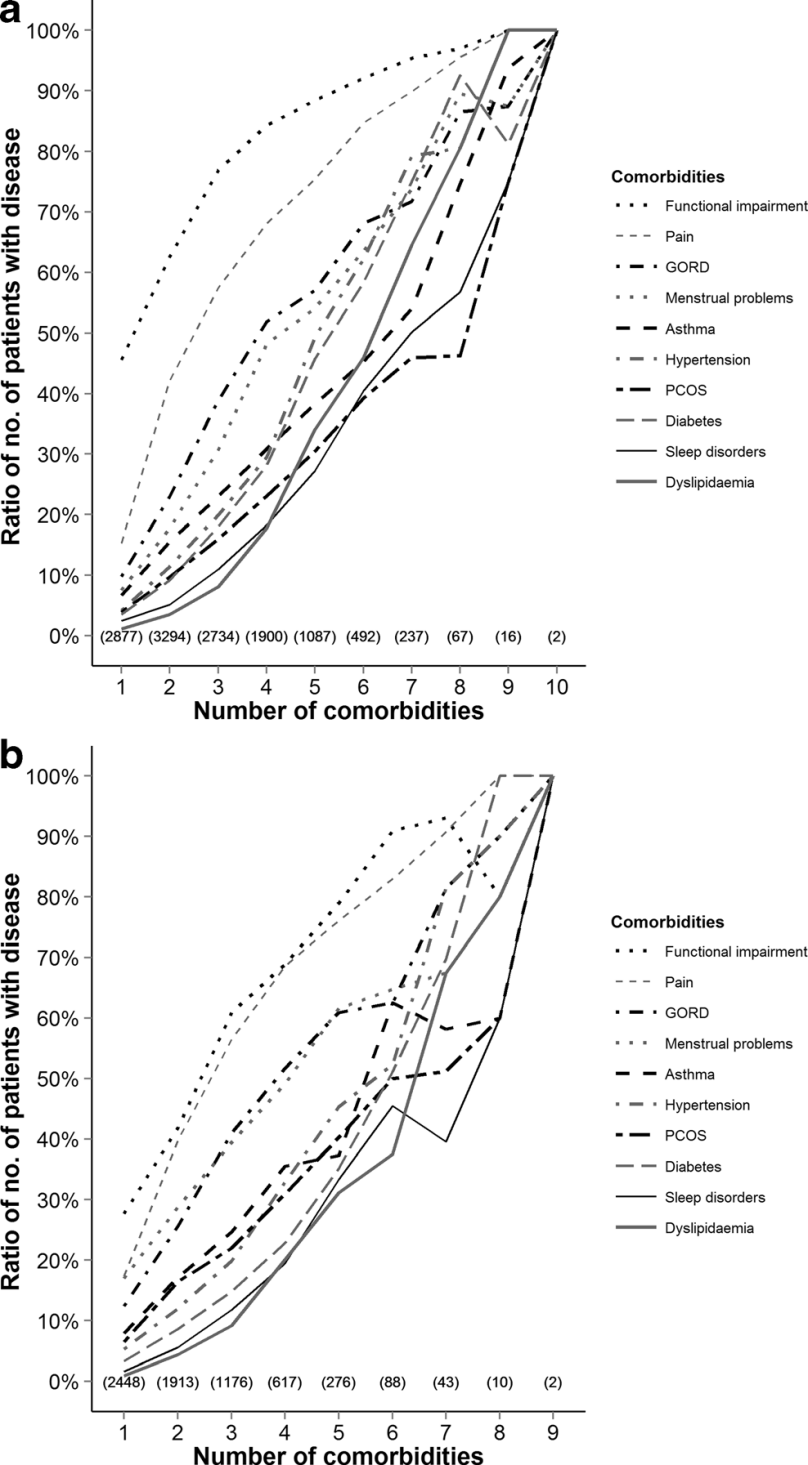
Relative contribution of each comorbidity before and after operation

Of those with a single comorbidity preoperatively, functional impairment was the most frequent (46 %). PCOS was the single comorbidity in only 4 % of these patients and menstrual dysfunction in 8 %. However, in patients with three comorbidities, menstrual dysfunction was more commonly seen: 16 % had PCOS, and 30 % had menstrual dysfunction (Fig. 3).

## Discussion

This study demonstrates that bariatric surgery is effective at improving factors that may underlie fertility and pregnancy outcomes: body weight, type 2 diabetes, menstrual dysfunction, and PCOS. Although baseline numbers may be questioned in view of missing data, our results demonstrate improvements in the prevalence of each comorbidity, even after application of sensitivity analyses.

The meta-analyses have demonstrated that bariatric surgery improves maternal health during pregnancy (including reduction in rates of gestational diabetes [11–13] and hypertensive disorders [11, 13] as well as fetal benefits such as a reduction in rates of macrosomia [11–13]). Few studies have assessed the effect of bariatric surgery on menstrual disorders (including PCOS), but those available demonstrate excellent results with 70–80 % achieving normal menstruation postoperatively [14, 15] although sample sizes are small. Future research should include a prospective cohort design to investigate whether the improvements in the comorbidities we have demonstrated also translate to improved fertility.

The dramatic effect on weight is in keeping with data on the overall bariatric surgery population [2]. A consensus view is that a reduction in body weight of 10 % is a realistic target that may help improve fertility [16]. Our data show that bariatric surgery is a very effective means to achieve such a target (87.3 % of patients achieved ≥10 % weight loss).

Our data gives an intimation of the relative importance to stakeholders of the comorbidities that lead to a decision for bariatric surgery. Our data confirm that type 2 diabetes is an important trigger for bariatric surgery [17] as it was overrepresented in the NBSR compared to the HSE cohort. However, only 3.5 % of patients with a single comorbidity had diabetes, suggesting that it is not necessarily taken as an indication on its own. A similar comparison to national data for PCOS and menstrual dysfunction could not be made as data in the national HSE cohort were not available for PCOS and menstrual dysfunction.

Of those with a single comorbidity, PCOS was recorded as frequently as diabetes (3.8 %) but almost twice as many had a record of menstrual dysfunction (8 %). Overall, our data demonstrate that PCOS is seen in one in six women having bariatric surgery and menstrual dysfunction is seen in almost one third of women.

The potential effect of bariatric surgery to improve fertility, whether as the primary aim of surgery or otherwise, highlights the importance of good preconception care—which is often suboptimal. In one study, 40 % of women were not aware of the recommendation to avoid pregnancy in the first 12–18 months and 30 % did not use any contraception in the 12 months after operation [18]. Adherence to these recommendations may be important to avoid poor neonatal outcomes from macronutrient or micronutrient deficiencies [19, 20].

In order to maximize the benefits of bariatric surgery on fertility and pregnancy outcomes, the potential complications of surgery must be identified and managed appropriately. A recent retrospective analysis suggested a possible increase in neonatal mortality in mothers who underwent bariatric surgery, but this did not control for comorbidities that are likely to be important confounders [12]. Nevertheless, there may be an increased risk of preterm delivery [13] and small-for-gestational-age newborns [11–13] that may be related to the type of procedure: Biliopancreatic diversion is much more likely to be associated with small-for-gestational-age neonates [19], while laparoscopic-assisted gastric bypass does not appear to increase the rate of small neonates [13]. Note that our data demonstrate that gastric bypass is the most common operation and that biliopancreatic diversion is rarely performed. Although included in the NBSR, intragastric balloon insertion may be considered a bariatric procedure rather than surgery per se. Our data showed that while these patients have similar demographics at baseline, the reductions in BMI were less (49.8 ± 18.1 to 45.7 ± 15.0 kg/m^2^) and improvements in the comorbidities were not seen. Indeed, the rates of different operations in female patients of childbearing age closely match those in the total NBSR cohort (Table 1). The similar rates for each operation raise the issue of whether this is appropriate or whether different operations should be offered to these women.

Open and timely access to bariatric surgery is important to optimize its benefits. Our data demonstrate that ethnic minorities are under-represented in the operated population, suggesting reduced access. This will require further investigation to explain possible cultural barriers and to prevent health inequalities in access to services.

The operated cohort was older than the eligible population of age-matched women. One interpretation for this is that there is a lead time from patients being eligible for surgery to having the operation. This delay is important as it falls around the key age for pregnancy success with a diminishing ovarian reserve. It has been demonstrated that before the age of 37, obesity has a significant negative impact on fertility, whereas in older women, this effect is outweighed by advancing age [21]. Thus, weight loss to improve fertility should ideally occur before this age [22]. That such a delay is happening may be due to delays in identification of eligible patients, delays in referral, and waiting lists for the operation itself. Clinicians should consider the benefits of operating earlier in young obese women, especially if fertility is a major concern.

The limitations of this study stem from limitations of the registry itself. Further information on preoperative childbearing history, parous state, and postoperative pregnancy would allow more direct conclusions to be drawn about the effects on fertility and pregnancy. Other data surrounding preconception care such as postoperative micronutrient supplementation or deficiency, contraceptive use, and hormonal supplementation are not recorded. It should also be noted that there is no site visit for the verification of the reported data. In the NBSR, the data is self-reported by each surgeon.

In summary, over half of all bariatric procedures are carried out on women of reproductive age. At least one in three of these women have menstrual dysfunction at baseline. Bariatric surgery improves factors that underlie fertility and pregnancy outcomes. A prospective study is required to demonstrate that this effect translates into a positive effect on pregnancy outcomes.
